# Polycythemia and chorea

**DOI:** 10.4103/0256-4947.51815

**Published:** 2009

**Authors:** Feroze Ahmad, Ravouf Asimi, Sakeena Rasool

**Affiliations:** a From the Department of Internal Medicine, Sher-i-Kashmir Institute of Medical Sciences, Soura, India; b From the Department of Neurology, Sher-i-Kashmir Institute of Medical Sciences, Soura, India; c From the Department of Medicine Government Medical College, Srinagar, India

**To the Editor:** Neurological manifestations of polycythemia occur frequently (50%-78%) and include headache, vertigo, stroke, visual symptoms, tinnitus, and paresthesia.^1^ Chorea, however, is a rare and infrequently reported complication of the disease (0.5%-5%).^2^–^4^ A 60-year-old male was admitted to our medical center following a month of involuntary, irregular and jerky movements of the limbs and abnormal involuntary tongue movements. These movements were random and fleeting from one part of the body to another causing difficulty in his day-to-day activities and social embarrassment. There was a history of heaviness in the head and continuous headache associated with mild visual blurring. There was no history of cognitive decline, strokes, drug or toxin exposure nor trauma or any lung disease. The patient was a non-smoker. There was no related family history. Examination revealed facial plethora and congested conjunctivae. There was no clubbing, splenomegaly or evidence of peripheral arterial or venous thromboembolism. His chest, cardiovascular and abdominal examinations were unremarkable. The neurological examination showed generalized chorieform movements involving the face and tongue. There was mild hypotonia of the limbs. Cognition was intact as evaluated by the Mini-Mental State Examination. Hemoglobin and hematocrit were increased, ranging from 17.0 g/dL to 19.2 g/dL and 56.9% to 65.8%, respectively. The WBC count was 5.1×10^3^/mL and the red cell count was 7.05×10^6^/mm^3^. The platelet count was normal. The red cell mass was increased to 38 mL/kg (normal, 26-34 mL/kg in men, 21-29 mL/kg in women) using the principal of isotope dilution by administering 5^1^ Crilabeled autologous red blood cells to the patient and sampling blood over a 2-hour period. Serum erythropoietin levels were mildly elevated. His arterial blood gas assessment showed a pH of 7.37, a PCO_2_ of 42.9 mm Hg, a PO_2_ of 62.6 mm Hg, an O_2_ saturation of 86.6%, and an HCO3 of 22.7 mm Hg. Red cell indices were a mean corpuscular volume of 93.3 fL, a mean corpuscular hemoglobin of 27.2 pg/cell, a mean corpuscular hemoglobin concentration of 30.1 g/dL. The erythrocyte sedimentation rate was 0 mm/h. The peripheral blood smear revealed no abnormal cell or acanthocytes. Renal, hepatic and other metabolic profiles were normal. The chest x-ray and echocardiogram were normal. Ultrasonography abdomen revealed no renal mass or organomegaly. The CT scan of brain was normal. Nerve conduction studies also were normal. The patient was diagnosed as having polycythemia chorea and was managed with phlebotomy (about 250 mL each) and a small dose of haloperidol. Improvement in the clinical picture was simultaneous with normalization of hemoglobin and packed cell volume. The erythrocyte sedimentation rate was 0 mm/h.

Polycythemia occurs predomnantly in males (3:2) but chorea secondary to polycythemia occurs predominantly in females. The prevalence is 1% to 2.5% of polycythemia patients.^4^ PC manifests predominantly after the age of 50 years, making polycythemia the first disorder to be considered in cases of so-called ‘senile’ chorea. PC is generalized, predominantly with involvement of faciolingual and brachial muscles, and is associated with muscular hypotonia. PC may last from periods of weeks to years, usually responds to haloperidol and venesection, but may persist, or recur with treatment, or remit spontaneously.^5^ The cause of the choreatic syndrome in polycythemia is presumably explained as a neostriatal hyperviscosity syndrome producing venous stasis, reduced brain blood flow and impaired tissue O_2_/glucose metabolism.^4^ The most important determinant of the viscosity of whole blood is the packed cell volume, and an inverse relationship can be shown between cerebral blood flow and packed cell volume.^5^ The state of dopaminergic hyperactivity is presumably enhanced by relatively increased neostriatal catecholestrogens. The cause of the choreatic syndrome in polycythaemia is presumably explained as a neostriatal hyperviscosity syndrome producing venous stasis, reduced brain blood flow and impaired tissular O_2_/glucose metabolism. The state of dopaminergic hyperactivity is presumably enhanced by relatively increased neostriatal catecholestrogens. The hypothesis of polycythaemic excess of dopamine-laden platelets releasing excess of dopamine in the neostriatum needs to be confirmed by laboratory evidence of platelet counts. It has been inferred from viscometric studies that red blood cell deformability might be reduced in iron deficiency,^6^ and the effect of iron-deficient red cell changes on whole blood viscosity has been assessed at a wide range of standardized packed cell volumes.^7^

In the treatment of chorea with polycythemia, venesection appears to be the initial method of choice. The observation that improvement in chorea may be independent of the erythrocyte count and hemoglobin level suggests that in some cases thrombocytosis is an important factor and that, in these cases, 32P or other bone marrow suppressing agents should also be given.^8^
